# Prehospital Early Warning Scores to Predict Mortality in Patients Using Ambulances

**DOI:** 10.1001/jamanetworkopen.2023.28128

**Published:** 2023-08-09

**Authors:** Tim Alex Lindskou, Logan Morgan Ward, Morten Breinholt Søvsø, Mads Lause Mogensen, Erika Frischknecht Christensen

**Affiliations:** 1 Centre for Prehospital and Emergency Research, Aalborg University Hospital and Department of Clinical Medicine, Aalborg University, Aalborg, Denmark; 2 Treat Systems ApS, Aalborg, Denmark; 3 Emergency Medical Services, North Denmark Region, Aalborg, Denmark

## Abstract

**Importance:**

Early warning scores (EWSs) are designed for in-hospital use but are widely used in the prehospital field, especially in select groups of patients potentially at high risk. To be useful for paramedics in daily prehospital clinical practice, evaluations are needed of the predictive value of EWSs based on first measured vital signs on scene in large cohorts covering unselected patients using ambulance services.

**Objective:**

To validate EWSs’ ability to predict mortality and intensive care unit (ICU) stay in an unselected cohort of adult patients who used ambulances.

**Design, Setting, and Participants:**

This prognostic study conducted a validation based on a cohort of adult patients (aged ≥18 years) who used ambulances in the North Denmark Region from July 1, 2016, to December 31, 2020. EWSs (National Early Warning Score 2 [NEWS2], modified NEWS score without temperature [mNEWS], Quick Sepsis Related Organ Failure Assessment [qSOFA], Rapid Emergency Triage and Treatment System [RETTS], and Danish Emergency Process Triage [DEPT]) were calculated using first vital signs measured by ambulance personnel. Data were analyzed from September 2022 through May 2023.

**Main Outcomes and Measures:**

The primary outcome was 30-day-mortality. Secondary outcomes were 1-day-mortality and ICU admission. Discrimination was assessed using area under the receiver operating characteristic curve (AUROC) and area under the precision recall curve (AUPRC).

**Results:**

There were 107 569 unique patients (52 650 females [48.9%]; median [IQR] age, 65 [45-77] years) from the entire cohort of 219 323 patients who used ambulance services, among whom 119 992 patients (54.7%) had called the Danish national emergency number. NEWS2, mNEWS, RETTS, and DEPT performed similarly concerning 30-day mortality (AUROC range, 0.67 [95% CI, 0.66-0.68] for DEPT to 0.68 [95% CI, 0.68-0.69] for mNEWS), while qSOFA had lower performance (AUROC, 0.59 [95% CI, 0.59-0.60]; *P* vs other scores < .001). All EWSs had low AUPRCs, ranging from 0.09 (95% CI, 0.09-0.09) for qSOFA to 0.14 (95% CI, 0.13-0.14) for mNEWS.. Concerning 1-day mortality and ICU admission NEWS2, mNEWS, RETTS, and DEPT performed similarly, with AUROCs ranging from 0.72 (95% CI, 0.71-0.73) for RETTS to 0.75 (95% CI, 0.74-0.76) for DEPT in 1-day mortality and 0.66 (95% CI, 0.65-0.67) for RETTS to 0.68 (95% CI, 0.67-0.69) for mNEWS in ICU admission, and all EWSs had low AUPRCs. These ranged from 0.02 (95% CI, 0.02-0.03) for qSOFA to 0.04 (95% CI, 0.04-0.04) for DEPT in 1-day mortality and 0.03 (95% CI, 0.03-0.03) for qSOFA to 0.05 (95% CI, 0.04-0.05) for DEPT in ICU admission.

**Conclusions and Relevance:**

This study found that EWSs in daily clinical use in emergency medical settings performed moderately in the prehospital field among unselected patients who used ambulances when assessed based on initial measurements of vital signs. These findings suggest the need of appropriate triage and early identification of patients at low and high risk with new and better EWSs also suitable for prehospital use.

## Key Points

**Question:**

How do early warning scores perform in predicting mortality and intensive care unit admission in adults using emergency medical services?

**Findings:**

In this prognostic study of 107 569 unique patients who used ambulances, usual early warning scores performed moderately in predicting mortality and intensive care unit admission as measured by area under the receiver operating characteristic curve. At typical operating points, there were high numbers of false negatives and false positives, suggesting risk of undertriage and overtriage.

**Meaning:**

These findings suggest that improved early warning scores may be needed for appropriate triage and early identification of patients in the prehospital setting.

## Teaser

This prognostic study investigates the performance of early warning scores in predicting 30-day mortality among patients who used ambulances.

## Introduction

Early prediction of serious outcomes is important in emergency care, the earlier, the better, ideally already when the patient is initially assessed on scene by paramedics. Originally, prognostic tools or early warning scores (EWSs), such as the National Early Warning Score (NEWS),^1^ based on vital signs were developed for in-hospital use to detect critical clinical deterioration early and initiate treatment, ultimately avoiding in-hospital cardiac arrest. Over the years, NEWS has been modified and other EWSs have been developed based at national or regional levels. Although studies of EWSs^1,2,3,4,5,6^ often show moderately high sensitivity and specificity, the low prevalence of patients with critical illness means that EWSs identify a considerable number of false-positive cases.^7^ The resulting overtriage may compromise health care professionals’ compliance, thereby reducing the clinical value of EWSs.^8^

EWSs have also been increasingly used in the prehospital field by emergency medical services (EMS), with varying results. A 2022 systematic review^2^ that included studies of different EWSs showed lower predictive accuracy in prehospital use compared with in-hospital use. The review included 7 prehospital studies on modest numbers of patients, ranging from a few hundred^3,4^ to approximately 30 000.^5,6^ Of the 7 studies, 5 studies were based on selected patient groups at high risk, such as patients treated by the advanced life support unit or physician-staffed units, representing the subset of patients with the most severe illness or injury among those with ambulance contact in the prehospital setting. Another review by Patel et al^9^ from 2018 showed similar results and was also based on small- to moderate-size studies. However, for EWSs to be useful for paramedics in daily clinical practice, studies examining the predictive value of EWSs in large patient cohorts covering unselected patients using EMS are needed.

Our objective was to validate the ability of existing EWSs to predict mortality and intensive care unit (ICU) stay in a large and unselected cohort of adult patients who used ambulance services. Our focus was on the first set of vital signs measured on scene as a proxy for patients’ initial state when first encountered by ambulance professionals.

## Methods

This prognostic study was a population-based validation study based on a historic consecutive cohort of adult patients who used ambulances. Findings are reported in accordance with the Transparent Reporting of a Multivariable Prediction Model for Individual Prognosis or Diagnosis (TRIPOD) reporting guideline. In Denmark, patient consent is required to access medical records or have medical records provided. When it is not possible to obtain patient consent, according to Danish legislation, the Danish Patient Safety Authority may waive this requirement and approve the handover of patient medical records. This was the case for this study.

### Setting

The North Denmark Region has approximately 550 000 inhabitants, corresponding to one-tenth of the Danish population, including urban and rural areas, and is considered representative for the Danish population.^10^ Each Danish citizen is assigned a unique identifier in the form of a Danish civil registration number. This number is used by public authorities, such as when citizens contact health care services.

In Denmark, patients can choose to call the national emergency number, 112, or in less severe cases, their general practitioner, the out-of-hours general practitioner on call, or the medical helpline, 1813 (Capital Region only).^11^ At the Emergency Medical Coordination Centre, which receives calls to the 112 number of a medical nature, health care professionals assess the situation and decide whether to dispatch an ambulance or another EMS vehicle. The center also dispatches ambulances on the request of general practitioners in and out of hours.

All ambulances use the same electronic prehospital medical record, containing patient data and measurements of vital signs. Automatically measured vital signs are transferred from the monitor in the ambulance to the medical record, and data are stored at a central server. Vital signs are measured when clinically relevant (eg, temperature measured in case of suspected infection), and acute treatment of the patient takes priority over registration of vital signs.

### Participants

We included patients aged 18 years or older using ambulance services in the North Denmark Region from July 1, 2016, to December 31, 2020. We excluded patients who had no Danish civil registration number (17 092 of 274 042 patients [6.2%]), whose medical record linked to more than 1 person, whose time of death was registered before the record-creation date, who had no vital signs recorded, and who received diagnoses concerning death at hospital arrival.

### Variables and Data Sources

We included 5 EWSs: the National Early Warning Score 2 (NEWS2; score range, 0-20),^2,12^ modified NEWS score without temperature (mNEWS; score range, 0-17), Quick Sepsis Related Organ Failure Assessment (qSOFA; score range, 0-2),^13^ Rapid Emergency Triage and Treatment System (RETTS; score range, 1-4),^14^ and Danish Emergency Process Triage (DEPT; score range, 1-4).^15^ These scores are based on different numbers and combinations of vital signs: respiratory rate, oxygen saturation, heart rate, systolic blood pressure, mental status, alert or not, and temperature.

To calculate EWSs, we retrieved data on prehospital vital signs from the prehospital electronic health record. Logistic data concerning ambulances were gathered from logistic systems. Data concerning sex, age, and possible date of death were obtained from the Danish Civil Registration System. Finally, administrative information on hospital visits (admission, discharge dates, and admitting department) and in-hospital diagnoses according to the *International Statistical Classification of Diseases and Related Health Problems, Tenth Revision* (*ICD-10*) were retrieved from the regional patient administrative system. Specific *ICD-10* diagnoses describing death were R092 respiratory arrest, R96 sudden death, R98 unattended death, R99 certain circumstances regarding death, R991 brain death according to the Danish Health Act §176, and R992 cardiac death according to the Danish Health Act §176. Patients receiving any of these diagnoses were excluded from the study. All data sources were linked using unique patient civil registration numbers. Hospital admissions were determined to be associated with an ambulance journey if the admission time was within 6 hours of the ambulance request.

**Figure 1.**
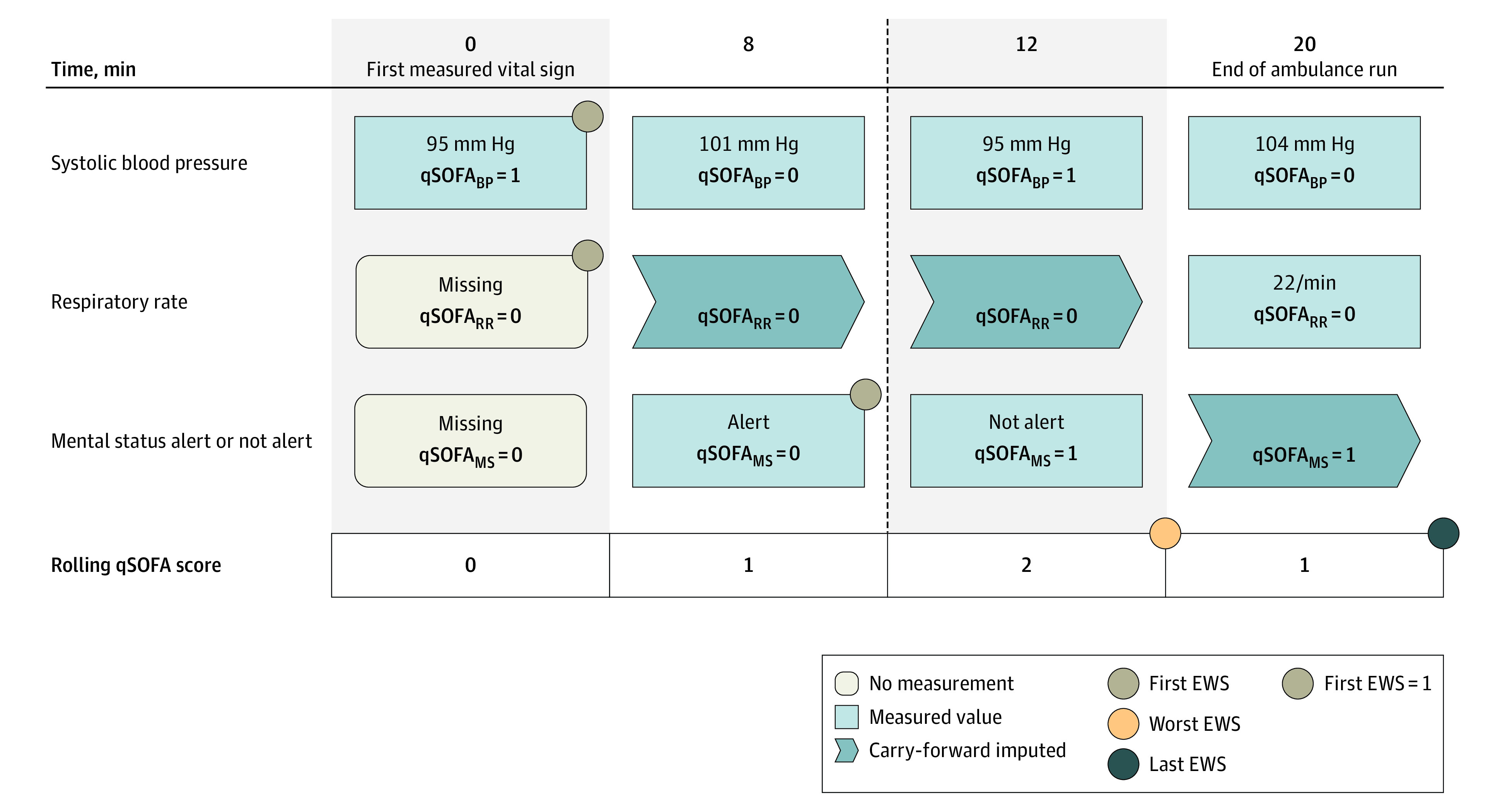
Scoring Concept For Measured Vital Signs To illustrate the scoring concept for first measured vital signs, a fictitious patient is presented. The patient has various measurements of vital signs recorded at 4 distinct times, from the first measurement recorded to 20 minutes later. The first early warning score (EWS) was calculated using systolic blood pressure (BP) as the first measured vital sign at minute 0. No respiratory rate (RR) was measured within the first 10 minutes and was therefore missing and imputed as normal. Finally, mental status (MS) was measured at minute 8. Summarized, the first measured vital sign EWS had a score of 1. Dotted vertical line indicates 10-minute cutoff used for determining first scores; qSOFA, Quick Sepsis Related Organ Failure Assessment.

For our primary analysis, EWSs were calculated using first measured vital signs, defined as vital signs measured within 10 minutes of the first vital sign registration. The first measurement of each parameter within 10 minutes after the first measurement was used to calculate the EWS (Figure 1). If any parameter was missing, it was imputed as normal, or within the score’s nonpathological or zero-scoring range.

As secondary analyses, we used last measured vital signs and the worst (ie, most severe) obtained score during the entire ambulance run. For each distinct time, if an individual vital sign was not measured, the most recent measurement for that vital sign was carried forward (forward imputation). To determine the highest score, the EWS was computed at each time and the highest calculated EWS score was selected. The last score was simply the last measured score during prehospital treatment (Figure 1).

### Outcomes

Our primary outcome was 30-day-mortality, and secondary outcomes were 1-day-mortality and admission to the ICU. Secondary outcomes were considered for each individual episode. For the 30-day mortality outcome, episodes were excluded from the analysis if they occurred in the 30-day follow-up period (30-day censored).

### Statistical Analysis

Receiver operating characteristic and precision-recall curves were plotted using standard clinical scores, and discrimination was assessed using area under the receiver operating characteristic curve (AUROC) and area under the precision recall curve (AUPRC). For standard or commonly used EWS threshold values (eTable 1 in Supplement 1), the performance at individual operating points was assessed using sensitivity, specificity, positive predictive value (PPV), negative predictive value (NPV), and the number of individuals needed to screen, defined as the number of false positives per true positive (1/PPV).

The Kaplan-Meier estimator was used to assess mortality. The Danish Civil Registration System does not specify time of death, so 1-day mortality was calculated from individuals with a date of death the same day as an ambulance journey or the following date.

Descriptive statistics are presented as number (percentage) for categorical variables and median (IQR) for nonnormally distributed continuous variables. Performance measures (AUROC, AUPRC, sensitivity, specificity, PPV, and NPV) are presented with 95% CIs, which were calculated using 1000 bootstrap resamples.

Differences in distributions of categorical and continuous variables were assessed using χ^2^ and Wilcoxon rank-sum tests, respectively. Differences in AUROC and AUPRC were computed via bootstrap. Sensitivity to missingness was assessed by comparing the overall discriminative performance of subgroups of patients with varying minimum levels of data completeness.

Data manipulation and calculation of statistical tests were carried out using Python programming language version 3.7.10 (Python Software Foundation), including packages scikit-learn and SciPy,^16,17^ and R statistical software version 3.6.1 (R Project for Statistical Computing). Survival analysis used the lifelines package.^18^ Statistical tests were 2-sided, and statistical significance was assumed at *P* < .05. Data were analyzed from September 2022 through May 2023.

## Results

**Figure 2.**
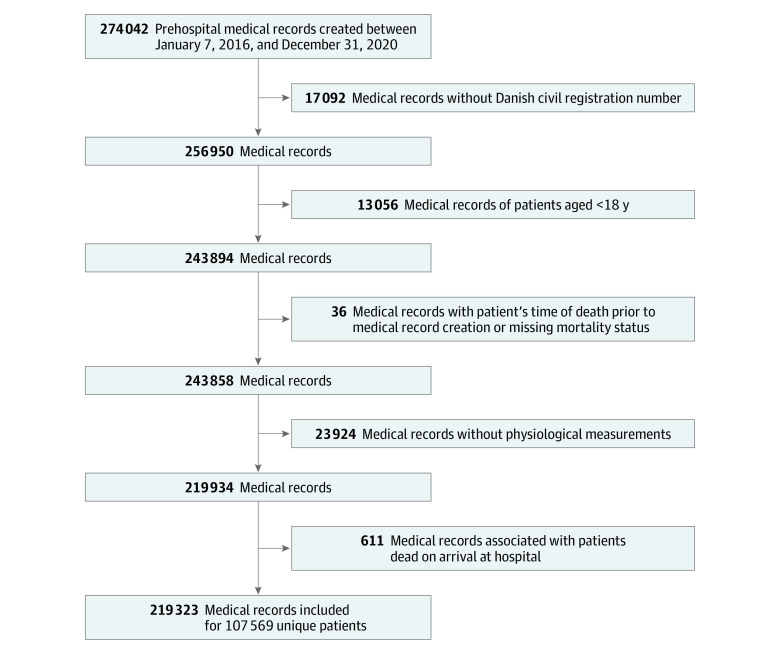
Flowchart of Data-Inclusion Process

There were 107 569 unique patients (52 650 females [48.9%]; median [IQR] age, 65 [45-77] years) from the entire cohort of 219 323 patients who used ambulance services (Figure 2; Table 1), among whom 119 992 patients (54.7%) called 112 and the remaining 99 331 patients (45.3%) had emergency services requested by general practitioners in and out of hours. Admission rates and mortality are shown in Table 1, and hospital diagnoses at the *ICD-10* level for patients brought to a hospital are shown in eFigure 1 in Supplement 1.

**Table 1.** Patient Characteristics

Characteristic	Patients, No. (%)		
	Total cohort (N = 219 323)	Unique individuals (N = 107 569)^a^	30-d Censored patients (n = 178 374)
Age, median (IQR), y	69 (52-80)	65 (45-77)	65 (50-80)
Sex			
Female	104 699 (47.7)	52 650 (48.9)	86 177 (48.3)
Male	114 624 (52.3)	54 919 (51.1)	92 197 (51.7)
Called emergency number	119 992 (54.7)	68 616 (63.8)	106 839 (59.9)
Total prehospital time, median (IQR), min	31 (18-46)	31 (18-46)	31 (18-46)
Admission			
Hospital	198 264 (90.4)	89 772 (83.5)	159 158 (89.2)
ICU	5044 (2.3)	2728 (2.5)	4373 (2.5)
Mortality, crude			
1 d	4119 (1.9)	1909 (1.8)	3308 (1.9)
3 d	6170 (2.8)	2532 (2.4)	4726 (2.6)
7 d	9344 (4.3)	3462 (3.2)	6865 (3.8)
30 d	18 650 (8.5)	6045 (5.6)	12 885 (7.2)

Abbreviation: ICU, intensive care unit.

^a^ Statistics for unique individuals use their first episode.

Table 2 and eTable 2 and eFigure 2 in Supplement 2 show vital signs registered in medical records, number of measurements, and completeness. As shown in eFigure 2 in Supplement 1, most patients had no or 1 missing vital sign and few had 5 or more missing vital signs. Considering that for the first measurement among the entire cohort, temperature was registered in 44 212 patients (20.2%), while other vital signs included in the scores were registered in a range from 139 533 patients (63.6%) for Glasgow Coma Scale to 179 965 patients (82.1%) for heart rate, EWS was based on medical records with at least 1 vital sign in more than 99% of patients (217 208 patients [99.0%]). Considering total prehospital time, vital sign recordings were more complete, with temperature registered in 91 553 patients (41.7%) and other vital signs registered in a range from 188 248 patients (85.8%) for respiratory rate to 203 802 patients (92.9%) for heart rate.

**Table 2.** Measurements and Clinical Scores for Entire Cohort Within First 10 Min

Measure	Medical records with measurement, No. (%) (N = 219 323)	Measurements, No.	Frequency, median (IQR)^a^	Distribution, median (IQR)
HR	179 965 (82.1)	725 681	4 (2-5)	86 (72-101)
SpO_2_	175 802 (80.2)	666 569	3 (2-5)	96 (94-98)
SBP	160 353 (73.1)	219 577	1 (1-2)	144 (124-162)
RR	144 261 (65.8)	186 704	1 (1-1)	18 (16-20)
GCS	139 533 (63.6)	163 588	1 (1-1)	15 (15-15)
Temperature	44 212 (20.2)	45 314	1 (1-1)	36.8 (36.6-37.6)
Clinical score^b^				
NEWS2	217 773 (99.3)	1 155 321	5 (3-7)	1 (0-3)
RETTS	217 773 (99.3)	1 155 321	5 (3-7)	1 (1-2)
mNEWS	217 526 (99.2)	1 119 120	5 (3-6)	1 (0-3)
DEPT	217 208 (99.0)	1 060 190	5 (3-6)	1 (1-2)
qSOFA	211 731 (96.5)	531 396	2 (2-3)	0 (0-0)

Abbreviations: DEPT, Danish Emergency Process Triage; GCS, Glasgow coma scale; HR, heart rate; mNEWS, modified NEWS score without temperature; NEWS2, National Early Warning Score 2; qSOFA, Quick Sepsis Related Organ Failure Assessment; RETTS, Rapid Emergency Triage and Treatment System; RR, respiratory rate; SBP, systolic blood pressure; SpO_2_, peripheral oxygen saturation.

^a^ Number of measurements per episode.

^b^ With at least 1 component measured.

**Figure 3.**
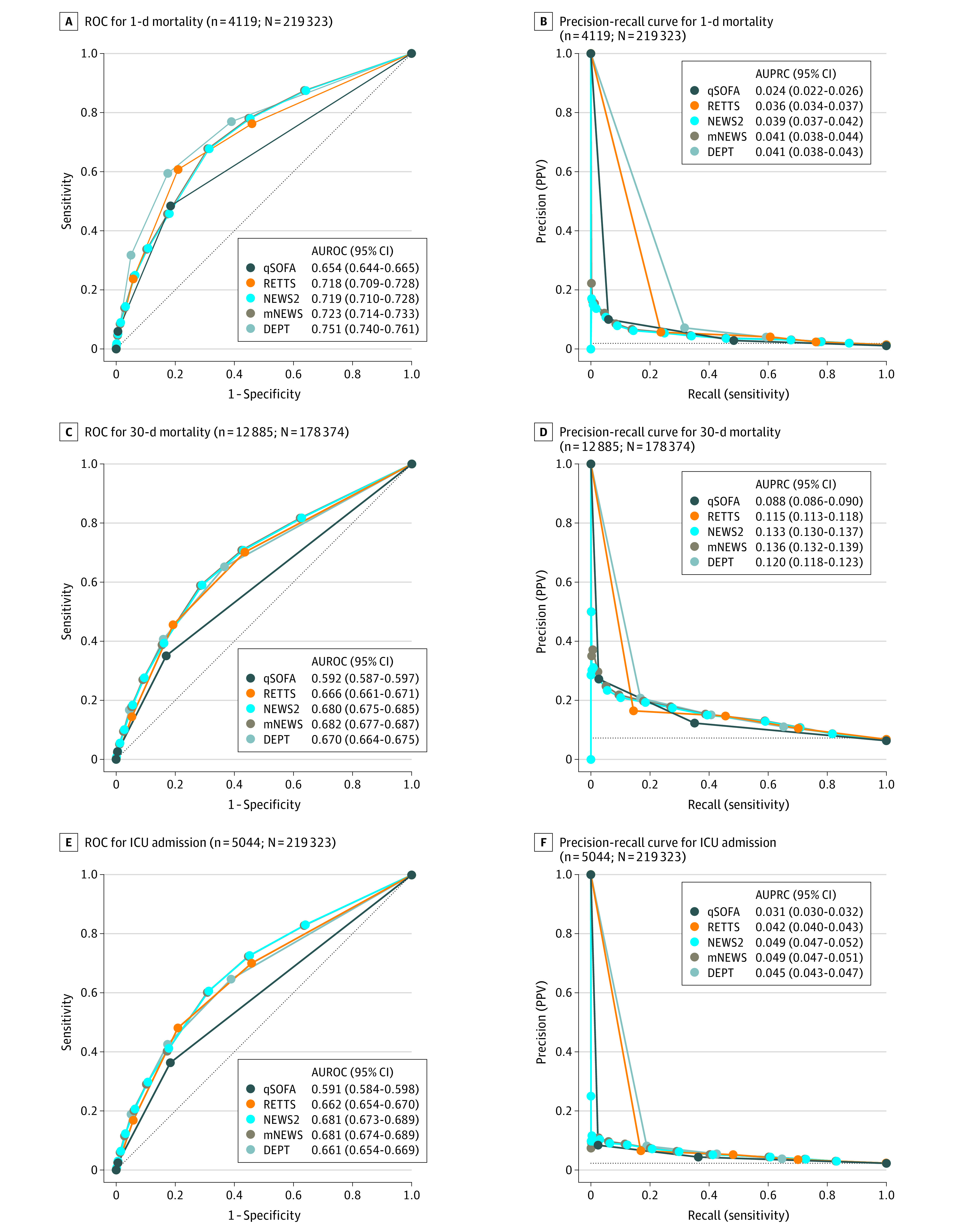
Receiver Operating Characteristic (ROC) and Precision Recall Curves for First Score Predictions AUPRC indicates area under the precision recall curve; AUROC, area under the receiver operating characteristic curve; DEPT, Danish Emergency Process Triage; mNEWS, modified NEWS score without temperature; NEWS2, National Early Warning Score 2; qSOFA, Quick Sepsis Related Organ Failure Assessment; RETTS, Rapid Emergency Triage and Treatment System. Diagonal and horizontal black dotted lines indicate lines of no discrimination; these are the curves that a purely random classifier would be expected to follow. For the ROC curve, this line is sensitivity (1 – specificity), and for the precision recall curve, this is precision (outcome rate).

NEWS2, mNEWS, RETTS, and DEPT performed similarly (Figure 3) concerning prediction of 30-day mortality, with AUROCs ranging from 0.67 (95% CI, 0.66-0.68) for DEPT to 0.68 (95% CI, 0.68-0.69) for mNEWS, while performance was lower for qSOFA, with an AUROC of 0.59 (95% CI, 0.59-0.60) (*P* vs other scores < .001). All EWSs had low AUPRCs, ranging from 0.09 (95% CI, 0.09-0.09) for qSOFA to 0.14 (95% CI, 0.13-0.14) for mNEWS. There was no significant difference in prediction of 1-day mortality for DEPT vs RETTS, NEWS2, or mNEWS or RETTS vs NEWS2 and mNEWS or 30-day mortality for DEPT vs RETTS. There was no significant difference concerning ICU admissions for NEWS vs mNEWS or DEPT vs RETTS. All other differences were statistically significant (Figure 3). For prediction of 1-day mortality, NEWS2, mNEWS, RETTS, and DEPT performed similarly to one another and better than for prediction of 30-day mortality, with AUROCs ranging from 0.72 (95% CI, 0.71-0.73) for RETTS to 0.75 (95% CI, 0.74-0.76) for DEPT, while prediction of ICU admission was similar to 30-day mortality, with AUROCs ranging from 0.66 (95% CI, 0.65-0.67) for RETTS to 0.68 (95% CI, 0.67-0.69) for mNEWS and low AUPRCs. These ranged from 0.02 (95% CI, 0.02-0.03) for qSOFA to 0.04 (95% CI, 0.04-0.04) for DEPT in 1-day mortality and 0.03 (95% CI, 0.03-0.03) for qSOFA to 0.05 (95% CI, 0.04-0.05) for DEPT in ICU admission. In sensitivity analyses, predictions based on highest or worst scores during the ambulance journey and last scores at ambulance arrival at the hospital performed significantly better (eFigures 3 and 4 in Supplement 1).

Performance metrics at standard or commonly used EWS threshold values are presented in eTable 3 in Supplement 1, showing moderate sensitivities, with the highest sensitivities in RETTS and DEPT. The highest sensitivities were found for 30-day mortality, and lower sensitivities were found for 1-day mortality. The number needed to screen (ie, false positives per true positives) at a score threshold of 2 or greater ranged from 2.7 (95% CI, 2.3-3.1) for qSOFA to 8.5 (95% CI, 8.3-8.7) for RETTS for 30-day mortality and 9.0 (95% CI, 7.8-10.8) for qSOFA to 41.6 (95% CI, 40.0-43.6) for DEPT for 1-day mortality. Overall, sensitivity increased and specificity decreased for analyses of highest or worst scores during the ambulance journey and last scores at ambulances arrival at the hospital (eFigures 3 and 4 and eTables 4 and 5 in Supplement 1).

Analyses of the association between missing data and score performance for EWSs calculated using vital measurements, worst vital measurements, and last vital measurements are presented in eFigures 5 through 7 in Supplement 1. For first scores, subgroups with a higher minimum level of completeness had higher AUROCs for all outcomes and all EWSs. There was no association between higher minimum completeness and higher AUROC for DEPT or RETTS in analyses using worst or last scores, but associations were seen for NEWS2, mNEWS, and qSOFA. Vital sign completeness was also associated with outcomes for first-measured and worst and last-measured values (eFigure 2 in Supplement 1). For worst and last-measured values, there was a stronger association between missingness and outcomes, but there were significantly fewer patients with a high degree of missing data.

## Discussion

In this large-scale, population-based prehospital prognostic study, RETTS, NEWS2, mNEWS, DEPT, and qSOFA scores all performed moderately in prediction of serious outcomes. Furthermore, all scores had a low PPV.

We found the scores to be of less value for early prediction of serious outcomes in the prehospital field than other studies,^3,4,5,6,8^ including other Scandinavian studies.^5,6,8^ In contrast to most of these studies, our study comprised the entire population of patients using ambulance services and not selected high-risk groups, such as patients treated by advanced units or doctor-staffed units. This may explain some differences from other studies due to the lower prevalence of patients with critical illness.^7^ The RETTS developed in Sweden and the DEPT, a Danish score based on a Swedish score, performed the best. This may be explained by the construction of scores given that NEWS2, mNEWS, and qSOFA present a different approach to triage compared with DEPT and RETTS. While DEPT and RETTS triage patient based on the worst single element of the score, NEWS2, mNEWS, and qSOFA are additive scores in which dysfunction across multiple criteria is required to trigger an alert. This was also reflected in the higher sensitivity when calculating NEWS2, mNEWS, and qSOFA based on worst or last values measured. Our focus was on scores based on initial measurements given that these represent the first available values for paramedics’ decision-making at the scene. In contrast, last scores are available just before the ambulance arrives at the hospital. Evaluating the worst score was a theoretical analysis to estimate the best performances of scores given that the worst score will not be practically applicable in daily clinical use.

In a 2019 systematic review, NEWS was reported to under-triage, with high number of deaths in hospital.^19^ The 5 EWSs in our study had neither sufficient sensitivity nor sufficient specificity in an unselected prehospital patient population and tended to undertriage and overtriage. While overtriage is necessary for the identification of patients with critical illness, the downside of a high number of false-positive cases is that it may lead to alert fatigue, eroding the utility of the score.^7,8^

The relatively low PPV of EWSs for identifying patients at high risk may be a problem owing to the steady increase in patients needing emergency treatment with the demographic development of greater numbers of older patients seen throughout the Western world. In this context, mortality prediction performance has been shown to improve when age is added to EWS, especially among individuals aged 80 years or older.^20^ Moreover, demographic changes in the population of patients needing emergency treatment have led to calls for a focus on nonconveyance of patients needing ambulance services.^9^ Thus, the ideal EWS for use in the EMS would not only identify patients at critically high risk, but also show better prediction of patients at very low risk to support paramedics’ decision-making about transferring to the hospital.

Current scores divide patients into large strata in which there is still significant variation among outcomes. Although validating existing scores for patients who have not yet been admitted to the hospital can assist in decision-making, development of a more nuanced score with specific targets may provide a superior classification. A well-calibrated probability score allows for rational decision-making based on known quantities.

### Limitations

This study has several limitations. The main limitation came from missing civil registration numbers, leading to exclusion of 6% of patients in the entire cohort and the risk of bias. The bias was probably not only in 1 direction given that it may be due to different cases, such as unknown death on scene, minor injury cases, or intoxication.

Missing measurements were also a limitation. In clinical use, clinical scoring systems intuitively count abnormal variables, whereas normal variables do not contribute to an increased score. Thus, considering missing measurements to be normal did not alter the score based on known measurements. Missing measurements could be due to ambulance personnel focusing on resuscitation or life-sustaining care as opposed to making diagnostic measurements. However, missing vital data may also be due to lack of clinical relevance, as reported among pediatric patients needing ambulance services, where missing data was associated with mild and more severe disease.^21^

Furthermore, our approach enabled us to include as many patients as possible but also entailed the risk of selection bias. However, the trade-off in choosing to exclude patients with few vital parameters would also introduce a selection bias due to differences in outcome rates across varying degrees of missingness.

The analysis of performance in the context of missing data showed higher AUROC and AUPRC values with more complete data, which suggests that missing data could not always be considered normal. In our analysis, high degrees of missing vital data (most patients had no or 1 missing vital sign, and few had ≥5 missing vital signs) were associated with higher mortality.

## Conclusions

This large prognostic study found that the EWS in daily clinical use in emergency medical settings performed moderately in the prehospital field among unselected patients receiving ambulance services when assessed based on initial measurements of vital signs. The need of appropriate triage and early identification of patients at low and high risk points toward new and better EWSs also suitable for prehospital use. Our next step is to investigate whether machine-learning methods may be associated with improved prediction and accuracy.

## Notes

Supplement 1. **eTable 1.** Overview of Early Warning Score Threshold Values **eTable 2.** Measurements and Clinical Scores for Entire Cohort for Total Prehospital Time **eTable 3.** Performance Metrics at Standard and Commonly Used Early Warning Score Threshold Values for First Score per Episode **eTable 4.** Performance Metrics at Standard and Commonly Used Early Warning Score Threshold Values for Worst Score per Episode **eTable 5.** Predictive Performance at Standard Thresholds for Last Early Warning Score per Episode **eFigure 1.** Distribution of Primary Admission Diagnosis for Patients Admitted to a Hospital **eFigure 2.** Association Between Vital Sign Completeness and Outcomes **eFigure 3.** Receiver Operating Characteristic and Precision-Recall Curves for Worst Scores’ Prediction of Outcomes **eFigure 4.** Receiver Operating Characteristic and Precision-Recall Curves for Last Scores’ Prediction of Outcomes **eFigure 5.** Sensitivity of Early Warning Score Predictive Ability to Minimum Number of Components Used to Calculate First Score **eFigure 6.** Sensitivity of Early Warning Score Predictive Ability to Minimum Number of Components Used to Calculate Worst Score **eFigure 7.** Sensitivity of Early Warning Score Predictive Ability to Minimum Number of Components Used to Calculate Last Score

Supplement 2.
Data Sharing Statement
