# Supplementary material for: Prehospital Early Warning Scores to Predict Mortality in Patients Using Ambulances
Source: JAMA Netw Open. 2023 Aug 9;6(8):e2328128. doi: 10.1001/jamanetworkopen.2023.28128 (PMC10413164; doi:10.1001/jamanetworkopen.2023.28128)
Supplement: Supplement 1. — eTable 1. Overview of Early Warning Score Threshold Values eTable 2. Measurements and Clinical Scores for Entire Cohort for Total Prehospital Time eTable 3. Performance Metrics at Standard and Commonly Used Early Warning Score Threshold Values for First Score per Episode eTable 4. Performance Metrics at Standard and Commonly Used Early Warning Score Threshold Values for Worst Score per Episode eTable 5. Predictive Performance at Standard Thresholds for Last Early Warning Score per Episode eFigure 1. Distribution of Primary Admission Diagnosis for Patients Admitted to a Hospital eFigure 2. Association Between Vital Sign Completeness and Outcomes eFigure 3. Receiver Operating Characteristic and Precision-Recall Curves for Worst Scores’ Prediction of Outcomes eFigure 4. Receiver Operating Characteristic and Precision-Recall Curves for Last Scores’ Prediction of Outcomes eFigure 5. Sensitivity of Early Warning Score Predictive Ability to Minimum Number of Components Used to Calculate First Score eFigure 6. Sensitivity of Early Warning Score Predictive Ability to Minimum Number of Components Used to Calculate Worst Score eFigure 7. Sensitivity of Early Warning Score Predictive Ability to Minimum Number of Components Used to Calculate Last Score [file jamanetwopen-e2328128-s001.pdf]

Supplemental Online Content

Lindskou TA, Ward LM, Søvsø MB, Mogensen ML, Christensen EF. Prehospital early warning scores to predict mortality in patients using ambulances. *JAMA Netw Open*. 2023;6(8):e2328128. doi:10.1001/jamanetworkopen.2023.28128

eTable 1. Overview of Early Warning Score Threshold Values

eTable 2. Measurements and Clinical Scores for Entire Cohort for Total Prehospital Time

eTable 3. Performance Metrics at Standard and Commonly Used Early Warning Score Threshold Values for First Score per Episode

eTable 4. Performance Metrics at Standard and Commonly Used Early Warning Score Threshold Values for Worst Score per Episode

eTable 5. Predictive Performance at Standard Thresholds for Last Early Warning Score per Episode

eFigure 1. Distribution of Primary Admission Diagnosis for Patients Admitted to a Hospital

eFigure 2. Association Between Vital Sign Completeness and Outcomes

eFigure 3. Receiver Operating Characteristic and Precision-Recall Curves for Worst Scores' Prediction of Outcomes

eFigure 4. Receiver Operating Characteristic and Precision-Recall Curves for Last Scores' Prediction of Outcomes

eFigure 5. Sensitivity of Early Warning Score Predictive Ability to Minimum Number of Components Used to Calculate First Score

eFigure 6. Sensitivity of Early Warning Score Predictive Ability to Minimum Number of Components Used to Calculate Worst Score

eFigure 7. Sensitivity of Early Warning Score Predictive Ability to Minimum Number of Components Used to Calculate Last Score

This supplemental material has been provided by the authors to give readers additional information about their work.

© 2023 Lindskou TA et al. *JAMA Network Open.*

eTable 1. Overview of EWS threshold values

EWS	NEWS2	mNEWS	qSOFA	RETTS	DEPT
Threshold values	score of 3 in any one category (red): medium<br><br>total score of 5-6: medium<br><br>total score of 7: high risk	score of 3 in any one category (red): medium<br><br>total score of 3: medium<br><br>total score of 4: medium-high<br><br>total score of 5: high	score of 1: low risk<br><br>score of ≥2: high risk (defined as threshold to require a full SOFA score to assess suspected sepsis)	2/yellow: less urgent<br><br>3/orange: urgent<br><br>4/red: life-threatening	2/yellow: less urgent<br><br>3/orange: urgent<br><br>4/red: life-threatening

© 2023 Lindskou TA et al. *JAMA Network Open.*

eTable 2. Measurements and clinical scores for the entire cohort for the total prehospital time

	Medical records with measurement, n (%)	Measurements, n	Frequency*, median [IQR]	Distribution, median [IQR] / n, (%)
Measured/recorded values				
HR	203,802 (92.9)	2,317,922	9 [6-14]	84 [71-99]
SpO2	201,786 (92.0)	2,087,934	8 [5-12]	96 [94-98]
SBP	198,428 (90.5)	695,049	3 [2-4]	137 [119-157]
RR	188,248 (85.8)	541,721	2 [1-3]	18 [16-22]
GCS	194,264 (88.6)	426,940	2 [1-3]	15 [15-15]
Temperature	91,553 (41.7)	101,910	1 [1-1]	36.8 [36.6-37.6]
*Clinical scores***				
NEWS2	219,043 (99.9)	3,414,411	13 [8-19]	1 [0-3]
RETTS	219,043 (99.9)	3,414,411	13 [8-19]	1 [1-2]
mNEWS	218,928 (99.8)	3,336,557	13 [8-18]	1 [0-3]
DEPT	218,830 (99.8)	3,129,007	12 [8-17]	1 [1-2]
qSOFA	217,556 (99.2)	1,579,671	6 [4-9]	0 [0-1]

* number of measurements per episode
**where at least one component was measured

© 2023 Lindskou TA et al. *JAMA Network Open.*

eTable 3. Performance metrics at standard/commonly used EWS threshold values for the first EWS score per episode. Metrics presented with 95% confidence interval.

Score		Outcome	N above threshold	Denom.	N true positive	Sensitivity	Specificity	Positive predictive value	Number needed to screen*	Negative predictive value
**NEWS**	Red**	M1	49912 (22.8)	219323	1905	0.462 [0.447-0.476]	0.777 [0.775-0.779]	0.038 [0.037-0.040]	25.2 [24.2-26.1]	0.987 [0.986-0.987]
		M30	39465 (22.1)	178374	5786	0.449 [0.439-0.456]	0.796 [0.795-0.798]	0.147 [0.143-0.149]	5.8 [5.7-6.0]	0.949 [0.948-0.950]
		ICU adm.	49912 (22.8)	219323	2494	0.494 [0.481-0.509]	0.779 [0.777-0.781]	0.050 [0.048-0.052]	19.0 [18.2-20.0]	0.985 [0.984-0.986]
	5	M1	23462 (10.7)	219323	1036	0.252 [0.237-0.263]	0.896 [0.895-0.897]	0.044 [0.042-0.047]	21.6 [20.2-23.0]	0.984 [0.984-0.985]
		M30	18440 (10.3)	178374	3208	0.249 [0.242-0.256]	0.908 [0.907-0.909]	0.174 [0.169-0.180]	4.7 [4.6-4.9]	0.939 [0.938-0.941]
		ICU adm.	23462 (10.7)	219323	1453	0.288 [0.277-0.299]	0.897 [0.896-0.898]	0.062 [0.059-0.065]	15.1 [14.4-15.9]	0.982 [0.981-0.982]
	7	M1	7049 (3.2)	219323	437	0.106 [0.098-0.116]	0.969 [0.969-0.970]	0.062 [0.057-0.067]	15.1 [14.0-16.8]	0.983 [0.982-0.983]
		M30	5606 (3.1)	178374	1172	0.091 [0.086-0.096]	0.973 [0.972-0.974]	0.209 [0.197-0.217]	3.8 [3.6-4.1]	0.932 [0.931-0.933]
		ICU adm.	7049 (3.2)	219323	601	0.119 [0.111-0.127]	0.970 [0.969-0.971]	0.085 [0.079-0.091]	10.7 [10.0-11.7]	0.979 [0.978-0.980]
**mNEWS**	Red**	M1	49817 (22.7)	219323	1904	0.462 [0.449-0.476]	0.777 [0.776-0.779]	0.038 [0.037-0.040]	25.2 [24.1-26.4]	0.987 [0.986-0.987]
		M30	39384 (22.1)	178374	5782	0.449 [0.440-0.456]	0.797 [0.795-0.798]	0.147 [0.144-0.150]	5.8 [5.7-6.0]	0.949 [0.948-0.950]
		ICU adm.	49817 (22.7)	219323	2488	0.493 [0.479-0.510]	0.779 [0.777-0.781]	0.050 [0.048-0.051]	19.0 [18.5-19.9]	0.985 [0.984-0.985]
	3	M1	66171 (30.2)	219323	2054	0.499 [0.486-0.514]	0.702 [0.700-0.704]	0.031 [0.030-0.033]	31.2 [29.7-32.3]	0.987 [0.986-0.987]
		M30	52250 (29.3)	178374	6810	0.529 [0.520-0.538]	0.725 [0.723-0.728]	0.130 [0.128-0.133]	6.7 [6.5-6.9]	0.952 [0.951-0.953]
		ICU adm.	66171 (30.2)	219323	2926	0.580 [0.569-0.595]	0.705 [0.703-0.707]	0.044 [0.043-0.046]	21.6 [21.0-22.3]	0.986 [0.986-0.987]
	4	M1	37322 (17.0)	219323	1384	0.336 [0.324-0.350]	0.833 [0.831-0.835]	0.037 [0.035-0.039]	26.0 [24.8-27.6]	0.985 [0.984-0.985]
		M30	29255 (16.4)	178374	4487	0.348 [0.339-0.356]	0.850 [0.849-0.852]	0.153 [0.149-0.158]	5.5 [5.3-5.7]	0.944 [0.942-0.945]
		ICU adm.	37322 (17.0)	219323	1957	0.388 [0.373-0.402]	0.835 [0.834-0.837]	0.052 [0.050-0.054]	18.1 [17.4-18.8]	0.983 [0.982-0.984]
	5	M1	22376 (10.2)	219323	1023	0.248 [0.235-0.263]	0.901 [0.899-0.902]	0.046 [0.043-0.048]	20.9 [19.8-22.3]	0.984 [0.984-0.985]
		M30	17511 (9.8)	178374	3123	0.242 [0.236-0.249]	0.913 [0.912-0.915]	0.178 [0.173-0.183]	4.6 [4.5-4.8]	0.939 [0.938-0.940]
		ICU adm.	22376 (10.2)	219323	1413	0.280 [0.267-0.291]	0.902 [0.901-0.903]	0.063 [0.060-0.067]	14.8 [14.0-15.8]	0.982 [0.981-0.982]
**qSOFA**	1	M1	37505 (17.1)	219323	1067	0.259 [0.246-0.272]	0.831 [0.829-0.832]	0.028 [0.027-0.030]	34.1 [32.8-36.6]	0.983 [0.983-0.984]
		M30	29620 (16.6)	178374	3636	0.282 [0.276-0.289]	0.843 [0.841-0.845]	0.123 [0.119-0.127]	7.1 [6.9-7.4]	0.938 [0.937-0.939]
		ICU adm.	37505 (17.1)	219323	1645	0.326 [0.312-0.338]	0.833 [0.831-0.834]	0.044 [0.042-0.046]	21.8 [20.9-22.8]	0.981 [0.981-0.982]
	2	M1	1311 (0.6)	219323	131	0.032 [0.025-0.038]	0.995 [0.994-0.995]	0.100 [0.084-0.114]	9.0 [7.8-10.8]	0.982 [0.981-0.982]
		M30	1004 (0.6)	178374	273	0.021 [0.019-0.023]	0.996 [0.995-0.996]	0.272 [0.251-0.301]	2.7 [2.3-3.1]	0.929 [0.928-0.930]
		ICU adm.	1311 (0.6)	219323	110	0.022 [0.018-0.025]	0.994 [0.994-0.995]	0.084 [0.071-0.101]	10.9 [8.9-13.0]	0.977 [0.977-0.978]
**DEPT**	2	M1	82607 (37.7)	219323	1939	0.471 [0.458-0.483]	0.625 [0.623-0.627]	0.023 [0.022-0.024]	41.6 [40.0-43.6]	0.984 [0.984-0.985]
		M30	65684 (36.8)	178374	7242	0.562 [0.553-0.570]	0.647 [0.644-0.649]	0.110 [0.108-0.112]	8.1 [7.9-8.3]	0.950 [0.949-0.951]

© 2023 Lindskou TA et al. *JAMA Network Open.*

		ICU adm.	82607 (37.7)	219323	3114	0.617 [0.605-0.629]	0.629 [0.627-0.631]	0.038 [0.036-0.039]	25.5 [24.7-26.3]	0.986 [0.985-0.986]
	3	M1	37612 (17.1)	219323	1497	0.363 [0.351-0.378]	0.832 [0.831-0.834]	0.040 [0.038-0.042]	24.1 [22.9-25.3]	0.986 [0.985-0.986]
		M30	29961 (16.8)	178374	4518	0.351 [0.344-0.360]	0.846 [0.845-0.848]	0.151 [0.147-0.155]	5.6 [5.5-5.8]	0.944 [0.942-0.945]
		ICU adm.	37612 (17.1)	219323	2050	0.406 [0.393-0.417]	0.834 [0.832-0.836]	0.055 [0.052-0.056]	17.3 [16.7-18.2]	0.984 [0.983-0.984]
	4	M1	11196 (5.1)	219323	800	0.194 [0.179-0.205]	0.952 [0.951-0.953]	0.071 [0.068-0.076]	13.0 [12.2-13.9]	0.984 [0.984-0.985]
		M30	8979 (5.0)	178374	1861	0.144 [0.140-0.151]	0.957 [0.956-0.958]	0.207 [0.198-0.215]	3.8 [3.7-4.0]	0.935 [0.934-0.936]
		ICU adm.	11196 (5.1)	219323	910	0.180 [0.172-0.193]	0.952 [0.951-0.953]	0.081 [0.076-0.086]	11.3 [10.6-12.2]	0.980 [0.980-0.981]
**RETTS**	2	M1	97646 (44.5)	219323	2317	0.563 [0.546-0.572]	0.557 [0.555-0.559]	0.024 [0.023-0.025]	41.1 [39.7-42.7]	0.985 [0.985-0.986]
		M30	77710 (43.6)	178374	8152	0.633 [0.623-0.640]	0.580 [0.578-0.582]	0.105 [0.103-0.107]	8.5 [8.3-8.7]	0.953 [0.952-0.954]
		ICU adm.	97646 (44.5)	219323	3413	0.677 [0.663-0.688]	0.560 [0.558-0.562]	0.035 [0.034-0.036]	27.6 [26.6-28.4]	0.987 [0.986-0.987]
	3	M1	45289 (20.6)	219323	1846	0.448 [0.433-0.463]	0.798 [0.796-0.800]	0.041 [0.039-0.042]	23.5 [22.7-24.5]	0.987 [0.986-0.987]
		M30	36071 (20.2)	178374	5292	0.411 [0.401-0.420]	0.814 [0.812-0.816]	0.147 [0.143-0.150]	5.8 [5.7-5.9]	0.947 [0.946-0.948]
		ICU adm.	45289 (20.6)	219323	2345	0.465 [0.451-0.478]	0.800 [0.798-0.801]	0.052 [0.050-0.054]	18.3 [17.6-19.0]	0.984 [0.984-0.985]
	4	M1	12700 (5.8)	219323	720	0.175 [0.163-0.183]	0.944 [0.943-0.945]	0.057 [0.053-0.061]	16.6 [15.4-18.3]	0.984 [0.983-0.984]
		M30	10202 (5.7)	178374	1676	0.130 [0.124-0.137]	0.948 [0.947-0.949]	0.164 [0.157-0.172]	5.1 [4.8-5.3]	0.933 [0.932-0.935]
		ICU adm.	12700 (5.8)	219323	821	0.163 [0.154-0.175]	0.945 [0.944-0.945]	0.065 [0.060-0.070]	14.5 [13.4-15.6]	0.980 [0.979-0.980]

*Number of screenings required to detect one true positive (equivalently, number false positives per true positive or 1/positive predictive value)
**Score of 3 for at least one parameter

© 2023 Lindskou TA et al. *JAMA Network Open.*

eTable 4. Performance metrics at standard/commonly used EWS threshold values for the worst EWS score per episode. Metrics presented with 95% confidence interval.

Score		Outcome	N above threshold	Denom.	N true positive	Sensitivity	Specificity	Positive predictive value	Number needed to screen*	Negative predictive value
**NEWS**	red**	M1	92310 (42.1)	219323	2669	0.648 [0.635-0.665]	0.583 [0.581-0.585]	0.029 [0.028-0.030]	33.6 [32.1-34.9]	0.989 [0.988-0.989]
		M30	74069 (41.5)	178374	8559	0.664 [0.656-0.673]	0.604 [0.602-0.606]	0.116 [0.113-0.118]	7.7 [7.5-7.8]	0.959 [0.957-0.960]
		ICU adm.	92310 (42.1)	219323	3703	0.734 [0.722-0.744]	0.586 [0.584-0.588]	0.040 [0.039-0.041]	23.9 [23.3-24.8]	0.989 [0.989-0.990]
	5	M1	57763 (26.3)	219323	2365	0.574 [0.559-0.589]	0.743 [0.741-0.744]	0.041 [0.039-0.043]	23.4 [22.4-24.2]	0.989 [0.989-0.990]
		M30	45902 (25.7)	178374	6904	0.536 [0.525-0.544]	0.764 [0.763-0.766]	0.150 [0.147-0.153]	5.6 [5.5-5.8]	0.955 [0.954-0.956]
		ICU adm.	57763 (26.3)	219323	3150	0.625 [0.608-0.635]	0.745 [0.744-0.747]	0.055 [0.053-0.057]	17.3 [16.6-18.1]	0.988 [0.988-0.989]
	7	M1	24396 (11.1)	219323	1666	0.404 [0.388-0.418]	0.894 [0.893-0.896]	0.068 [0.066-0.072]	13.6 [12.8-14.1]	0.987 [0.987-0.988]
		M30	19336 (10.8)	178374	4098	0.318 [0.310-0.325]	0.908 [0.907-0.909]	0.212 [0.207-0.217]	3.7 [3.6-3.9]	0.945 [0.944-0.946]
		ICU adm.	24396 (11.1)	219323	2008	0.398 [0.385-0.412]	0.896 [0.894-0.897]	0.082 [0.079-0.085]	11.1 [10.7-11.6]	0.984 [0.984-0.985]
**mNEWS**	red**	M1	92186 (42.0)	219323	2668	0.648 [0.635-0.665]	0.584 [0.582-0.586]	0.029 [0.028-0.030]	33.6 [32.2-34.9]	0.989 [0.988-0.989]
		M30	73962 (41.5)	178374	8557	0.664 [0.655-0.672]	0.605 [0.603-0.607]	0.116 [0.113-0.118]	7.6 [7.5-7.8]	0.959 [0.957-0.960]
		ICU adm.	92186 (42.0)	219323	3697	0.733 [0.722-0.744]	0.587 [0.585-0.589]	0.040 [0.039-0.041]	23.9 [23.3-24.8]	0.989 [0.989-0.990]
	3	M1	123040 (56.1)	219323	3740	0.908 [0.898-0.916]	0.446 [0.443-0.447]	0.030 [0.030-0.031]	31.9 [30.8-33.0]	0.996 [0.996-0.996]
		M30	98877 (55.4)	178374	10765	0.835 [0.829-0.840]	0.468 [0.465-0.470]	0.109 [0.107-0.111]	8.2 [8.0-8.4]	0.973 [0.972-0.974]
		ICU adm.	123040 (56.1)	219323	4301	0.853 [0.844-0.863]	0.446 [0.444-0.448]	0.035 [0.034-0.036]	27.6 [26.7-28.3]	0.992 [0.992-0.993]
	4	M1	82545 (37.6)	219323	2614	0.635 [0.619-0.650]	0.629 [0.627-0.630]	0.032 [0.031-0.033]	30.6 [29.4-32.1]	0.989 [0.988-0.989]
		M30	65865 (36.9)	178374	8340	0.647 [0.639-0.654]	0.652 [0.650-0.655]	0.127 [0.125-0.129]	6.9 [6.7-7.1]	0.960 [0.959-0.961]
		ICU adm.	82545 (37.6)	219323	3709	0.735 [0.723-0.747]	0.632 [0.630-0.634]	0.045 [0.044-0.046]	21.3 [20.5-22.0]	0.990 [0.990-0.991]
	5	M1	55884 (25.5)	219323	2355	0.572 [0.558-0.586]	0.751 [0.750-0.753]	0.042 [0.040-0.044]	22.7 [21.9-23.7]	0.989 [0.989-0.990]
		M30	44283 (24.8)	178374	6812	0.529 [0.517-0.538]	0.774 [0.771-0.776]	0.154 [0.150-0.157]	5.5 [5.4-5.6]	0.955 [0.954-0.956]
		ICU adm.	55884 (25.5)	219323	3117	0.618 [0.606-0.628]	0.754 [0.752-0.756]	0.056 [0.054-0.058]	16.9 [16.3-17.5]	0.988 [0.988-0.989]
**qSOFA**	1	M1	76408 (34.8)	219323	3252	0.790 [0.779-0.801]	0.660 [0.658-0.662]	0.043 [0.041-0.044]	22.5 [21.8-23.2]	0.994 [0.994-0.994]
		M30	60761 (34.1)	178374	8177	0.635 [0.625-0.644]	0.682 [0.680-0.684]	0.135 [0.132-0.137]	6.4 [6.3-6.6]	0.960 [0.959-0.961]
		ICU adm.	76408 (34.8)	219323	3402	0.674 [0.662-0.686]	0.659 [0.657-0.661]	0.045 [0.043-0.046]	21.5 [20.8-22.2]	0.989 [0.988-0.989]
	2	M1	12741 (5.8)	219323	1089	0.264 [0.253-0.280]	0.946 [0.945-0.947]	0.085 [0.081-0.090]	10.7 [10.1-11.2]	0.985 [0.985-0.986]
		M30	9918 (5.6)	178374	2446	0.190 [0.183-0.198]	0.955 [0.954-0.956]	0.247 [0.237-0.255]	3.1 [2.9-3.2]	0.938 [0.937-0.939]
		ICU adm.	12741 (5.8)	219323	1137	0.225 [0.213-0.236]	0.946 [0.945-0.947]	0.089 [0.085-0.094]	10.2 [9.6-11.0]	0.981 [0.981-0.982]
**DEPT**	2	M1	145230 (66.2)	219323	3651	0.886 [0.875-0.895]	0.342 [0.340-0.344]	0.025 [0.024-0.026]	38.8 [37.7-39.9]	0.994 [0.993-0.994]
		M30	117413 (65.8)	178374	11080	0.860 [0.854-0.866]	0.357 [0.355-0.360]	0.094 [0.093-0.096]	9.6 [9.4-9.8]	0.970 [0.969-0.972]

© 2023 Lindskou TA et al. *JAMA Network Open.*

		ICU adm.	145230 (66.2)	219323	4472	0.887 [0.879-0.894]	0.343 [0.341-0.345]	0.031 [0.030-0.032]	31.5 [30.7-32.4]	0.992 [0.992-0.993]
	3	M1	75329 (34.3)	219323	3408	0.827 [0.817-0.838]	0.666 [0.664-0.668]	0.045 [0.044-0.047]	21.1 [20.3-21.8]	0.995 [0.995-0.995]
		M30	60502 (33.9)	178374	8495	0.659 [0.652-0.667]	0.686 [0.684-0.688]	0.140 [0.138-0.143]	6.1 [6.0-6.2]	0.963 [0.962-0.964]
		ICU adm.	75329 (34.3)	219323	3499	0.694 [0.683-0.708]	0.665 [0.663-0.667]	0.046 [0.045-0.048]	20.5 [19.7-21.3]	0.989 [0.989-0.990]
	4	M1	27627 (12.6)	219323	2815	0.683 [0.668-0.695]	0.885 [0.883-0.886]	0.102 [0.098-0.105]	8.8 [8.5-9.1]	0.993 [0.993-0.994]
		M30	22369 (12.5)	178374	5022	0.390 [0.383-0.396]	0.895 [0.894-0.896]	0.225 [0.220-0.230]	3.5 [3.4-3.6]	0.950 [0.949-0.951]
		ICU adm.	27627 (12.6)	219323	2071	0.411 [0.398-0.424]	0.881 [0.879-0.882]	0.075 [0.072-0.078]	12.3 [11.8-13.0]	0.984 [0.984-0.985]
**RETTS**	2	M1	164348 (74.9)	219323	3814	0.926 [0.918-0.933]	0.254 [0.252-0.256]	0.023 [0.023-0.024]	42.1 [40.8-43.9]	0.994 [0.994-0.995]
		M30	133284 (74.7)	178374	11612	0.901 [0.896-0.906]	0.265 [0.262-0.266]	0.087 [0.086-0.089]	10.5 [10.3-10.7]	0.972 [0.970-0.973]
		ICU adm.	164348 (74.9)	219323	4669	0.926 [0.920-0.933]	0.255 [0.253-0.257]	0.028 [0.028-0.029]	34.2 [33.3-35.0]	0.993 [0.992-0.994]
	3	M1	83596 (38.1)	219323	3555	0.863 [0.852-0.873]	0.628 [0.626-0.630]	0.043 [0.041-0.044]	22.5 [21.8-23.4]	0.996 [0.996-0.996]
		M30	67323 (37.7)	178374	8859	0.688 [0.678-0.697]	0.647 [0.645-0.649]	0.132 [0.130-0.134]	6.6 [6.5-6.8]	0.964 [0.962-0.965]
		ICU adm.	83596 (38.1)	219323	3603	0.714 [0.701-0.726]	0.627 [0.625-0.628]	0.043 [0.042-0.044]	22.2 [21.6-23.1]	0.989 [0.989-0.990]
	4	M1	34881 (15.9)	219323	2874	0.698 [0.682-0.711]	0.851 [0.850-0.853]	0.082 [0.079-0.086]	11.1 [10.6-11.5]	0.993 [0.993-0.994]
		M30	28149 (15.8)	178374	5233	0.406 [0.399-0.415]	0.862 [0.860-0.863]	0.186 [0.182-0.191]	4.4 [4.2-4.7]	0.949 [0.948-0.950]
		ICU adm.	34881 (15.9)	219323	2172	0.431 [0.416-0.443]	0.847 [0.846-0.849]	0.062 [0.060-0.065]	15.1 [14.4-15.7]	0.984 [0.984-0.985]

*Number of screenings required to detect one true positive (equivalently, number false positives per true positive or 1/positive predictive value)
**Score of 3 for at least one parameter

© 2023 Lindskou TA et al. *JAMA Network Open.*

eTable 5. Predictive performance at standard thresholds for last EWS per episode

Score		Outcome	N above threshold	Denom.	N true positive	Sensitivity	Specificity	Positive predictive value	Number needed to screen*	Negative predictive value
**NEWS**	red**	M1	40384 (18.4)	219323	1994	0.484 [0.472-0.497]	0.822 [0.820-0.823]	0.049 [0.048-0.051]	19.3 [18.5-20.3]	0.988 [0.988-0.989]
		M30	31815 (17.8)	178374	5188	0.403 [0.395-0.411]	0.839 [0.838-0.840]	0.163 [0.160-0.168]	5.1 [5.0-5.3]	0.947 [0.946-0.949]
		ICU adm.	40384 (18.4)	219323	2194	0.435 [0.423-0.448]	0.822 [0.820-0.824]	0.054 [0.052-0.056]	17.4 [16.8-18.1]	0.984 [0.984-0.985]
	5	M1	29086 (13.3)	219323	1892	0.459 [0.445-0.474]	0.874 [0.872-0.875]	0.065 [0.063-0.068]	14.4 [13.8-15.0]	0.988 [0.988-0.989]
		M30	22845 (12.8)	178374	4648	0.361 [0.352-0.369]	0.890 [0.888-0.892]	0.203 [0.199-0.209]	3.9 [3.8-4.0]	0.947 [0.946-0.948]
		ICU adm.	29086 (13.3)	219323	2110	0.418 [0.406-0.431]	0.874 [0.873-0.875]	0.073 [0.070-0.075]	12.8 [12.3-13.4]	0.985 [0.984-0.985]
	7	M1	10454 (4.8)	219323	1149	0.279 [0.266-0.294]	0.957 [0.956-0.957]	0.110 [0.104-0.115]	8.1 [7.7-8.5]	0.986 [0.985-0.986]
		M30	8220 (4.6)	178374	2364	0.183 [0.177-0.190]	0.965 [0.964-0.965]	0.288 [0.279-0.296]	2.5 [2.4-2.6]	0.938 [0.937-0.939]
		ICU adm.	10454 (4.8)	219323	1067	0.212 [0.200-0.224]	0.956 [0.955-0.957]	0.102 [0.097-0.107]	8.8 [8.3-9.3]	0.981 [0.980-0.982]
**mNEWS**	red**	M1	40229 (18.3)	219323	1990	0.483 [0.470-0.500]	0.822 [0.821-0.824]	0.049 [0.048-0.051]	19.2 [18.5-20.1]	0.988 [0.988-0.989]
		M30	31678 (17.8)	178374	5177	0.402 [0.394-0.409]	0.840 [0.838-0.841]	0.163 [0.160-0.168]	5.1 [5.0-5.3]	0.947 [0.946-0.948]
		ICU adm.	40229 (18.3)	219323	2176	0.431 [0.417-0.441]	0.822 [0.821-0.824]	0.054 [0.052-0.056]	17.5 [16.8-18.3]	0.984 [0.983-0.985
	3	M1	73197 (33.4)	219323	3434	0.834 [0.822-0.846]	0.676 [0.674-0.678]	0.047 [0.045-0.048]	20.3 [19.7-21.0]	0.995 [0.995-0.996]
		M30	57857 (32.4)	178374	8649	0.671 [0.663-0.678]	0.703 [0.701-0.704]	0.149 [0.147-0.153]	5.7 [5.6-5.8]	0.965 [0.964-0.966]
		ICU adm.	73197 (33.4)	219323	3404	0.675 [0.662-0.689]	0.674 [0.672-0.676]	0.047 [0.045-0.048]	20.5 [19.9-21.1]	0.989 [0.988-0.989]
	4	M1	43572 (19.9)	219323	2164	0.525 [0.512-0.540]	0.808 [0.806-0.809]	0.050 [0.048-0.051]	19.1 [18.5-20.3]	0.989 [0.988-0.989]
		M30	34149 (19.1)	178374	5930	0.460 [0.453-0.468]	0.829 [0.827-0.831]	0.174 [0.170-0.177]	4.8 [4.6-4.9]	0.952 [0.951-0.953]
		ICU adm.	43572 (19.9)	219323	2600	0.515 [0.501-0.529]	0.809 [0.808-0.810]	0.060 [0.058-0.061]	15.8 [15.3-16.5]	0.986 [0.986-0.987]
	5	M1	27076 (12.3)	219323	1869	0.454 [0.438-0.467]	0.883 [0.882-0.884]	0.069 [0.066-0.072]	13.5 [12.8-14.2]	0.988 [0.988-0.989]
		M30	21144 (11.9)	178374	4503	0.349 [0.341-0.357]	0.899 [0.898-0.901]	0.213 [0.208-0.219]	3.7 [3.6-3.8]	0.947 [0.946-0.948]
		ICU adm.	27076 (12.3)	219323	2027	0.402 [0.387-0.415]	0.883 [0.882-0.885]	0.075 [0.072-0.078]	12.4 [11.8-13.0]	0.984 [0.984-0.985]
**qSOFA**	1	M1	53944 (24.6)	219323	3017	0.732 [0.717-0.746]	0.763 [0.761-0.765]	0.056 [0.054-0.058]	16.9 [16.2-17.4]	0.993 [0.993-0.994]
		M30	42365 (23.8)	178374	7118	0.552 [0.545-0.560]	0.787 [0.785-0.789]	0.168 [0.165-0.172]	5.0 [4.8-5.1]	0.958 [0.957-0.959]
		ICU adm.	53944 (24.6)	219323	2838	0.563 [0.550-0.578]	0.761 [0.760-0.763]	0.053 [0.051-0.055]	18.0 [17.3-18.6]	0.987 [0.986-0.987]
	2	M1	7136 (3.3)	219323	769	0.187 [0.174-0.198]	0.970 [0.970-0.971]	0.108 [0.101-0.114]	8.3 [7.8-9.0]	0.984 [0.984-0.985]
		M30	5502 (3.1)	178374	1628	0.126 [0.120-0.132]	0.977 [0.976-0.977]	0.296 [0.284-0.309]	2.4 [2.2-2.5]	0.935 [0.934-0.936]
		ICU adm.	7136 (3.3)	219323	684	0.136 [0.127-0.143]	0.970 [0.969-0.971]	0.096 [0.090-0.102]	9.4 [8.8-10.2]	0.979 [0.979-0.980]
**DEPT**	2	M1	78447 (35.8)	219323	3227	0.783 [0.771-0.799]	0.650 [0.649-0.653]	0.041 [0.040-0.042]	23.3 [22.7-24.1]	0.994 [0.993-0.994]
		M30	63033 (35.3)	178374	8018	0.622 [0.615-0.631]	0.668 [0.666-0.670]	0.127 [0.125-0.130]	6.9 [6.7-7.0]	0.958 [0.956-0.959]
		ICU adm.	78447 (35.8)	219323	3244	0.643 [0.631-0.657]	0.649 [0.647-0.651]	0.041 [0.040-0.043]	23.2 [22.5-24.1]	0.987 [0.987-0.988]

© 2023 Lindskou TA et al. *JAMA Network Open.*

	3	M1	29578 (13.5)	219323	2783	0.676 [0.662-0.687]	0.875 [0.874-0.877]	0.094 [0.091-0.097]	9.6 [9.3-10.0]	0.993 [0.993-0.993]
		M30	23511 (13.2)	178374	5090	0.395 [0.387-0.402]	0.889 [0.887-0.890]	0.216 [0.212-0.222]	3.6 [3.5-3.7]	0.950 [0.949-0.951]
		ICU adm.	29578 (13.5)	219323	1967	0.390 [0.377-0.404]	0.871 [0.870-0.873]	0.067 [0.064-0.069]	14.0 [13.4-14.7]	0.984 [0.983-0.984]
	4	M1	8965 (4.1)	219323	2137	0.519 [0.501-0.535]	0.968 [0.968-0.969]	0.238 [0.231-0.247]	3.2 [3.1-3.3]	0.991 [0.990-0.991]
		M30	7383 (4.1)	178374	2784	0.216 [0.209-0.224]	0.972 [0.971-0.973]	0.377 [0.365-0.388]	1.7 [1.6-1.8]	0.941 [0.940-0.942]
		ICU adm.	8965 (4.1)	219323	872	0.173 [0.161-0.184]	0.962 [0.962-0.963]	0.097 [0.092-0.105]	9.3 [8.5-9.9]	0.980 [0.980-0.981]
**RETTS**	2	M1	90308 (41.2)	219323	3372	0.819 [0.808-0.830]	0.596 [0.594-0.598]	0.037 [0.036-0.039]	25.8 [24.7-26.7]	0.994 [0.994-0.995]
		M30	72510 (40.7)	178374	8540	0.663 [0.655-0.671]	0.613 [0.611-0.616]	0.118 [0.115-0.120]	7.5 [7.3-7.6]	0.959 [0.958-0.960]
		ICU adm.	90308 (41.2)	219323	3404	0.675 [0.661-0.690]	0.594 [0.592-0.596]	0.038 [0.037-0.039]	25.5 [24.8-26.5]	0.987 [0.987-0.988]
	3	M1	27923 (12.7)	219323	2759	0.670 [0.653-0.681]	0.883 [0.882-0.884]	0.099 [0.096-0.102]	9.1 [8.8-9.5]	0.993 [0.992-0.993]
		M30	22204 (12.4)	178374	4862	0.377 [0.369-0.383]	0.895 [0.894-0.897]	0.219 [0.214-0.224]	3.6 [3.5-3.7]	0.949 [0.948-0.950]
		ICU adm.	27923 (12.7)	219323	1851	0.367 [0.355-0.382]	0.878 [0.877-0.880]	0.066 [0.063-0.069]	14.1 [13.5-14.7]	0.983 [0.983-0.984]
	4	M1	11585 (5.3)	219323	2149	0.522 [0.506-0.536]	0.956 [0.955-0.957]	0.185 [0.179-0.192]	4.4 [4.2-4.6]	0.991 [0.990-0.991]
		M30	9411 (5.3)	178374	2986	0.232 [0.226-0.241]	0.961 [0.960-0.962]	0.317 [0.309-0.327]	2.2 [2.1-2.2]	0.941 [0.940-0.943]
		ICU adm.	11585 (5.3)	219323	996	0.197 [0.187-0.209]	0.951 [0.950-0.951]	0.086 [0.082-0.091]	10.6 [10.0-11.3]	0.981 [0.980-0.981]

*Number of screenings required to detect one true positive (equivalently, number false positives per true positive or 1/positive predictive value)
**Score of 3 for at least one parameter

© 2023 Lindskou TA et al. *JAMA Network Open.*

eFigure 1. Distribution of primary admission diagnosis for patients admitted to a hospital

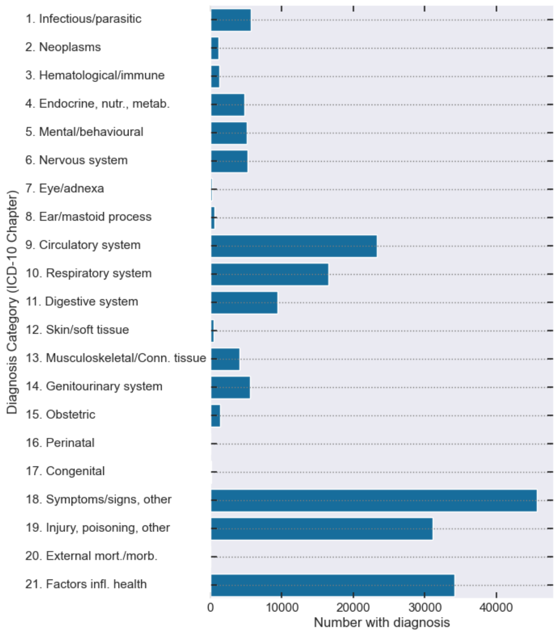

© 2023 Lindskou TA et al. *JAMA Network Open.*

eFigure 2. Association between vital sign completeness and outcomes

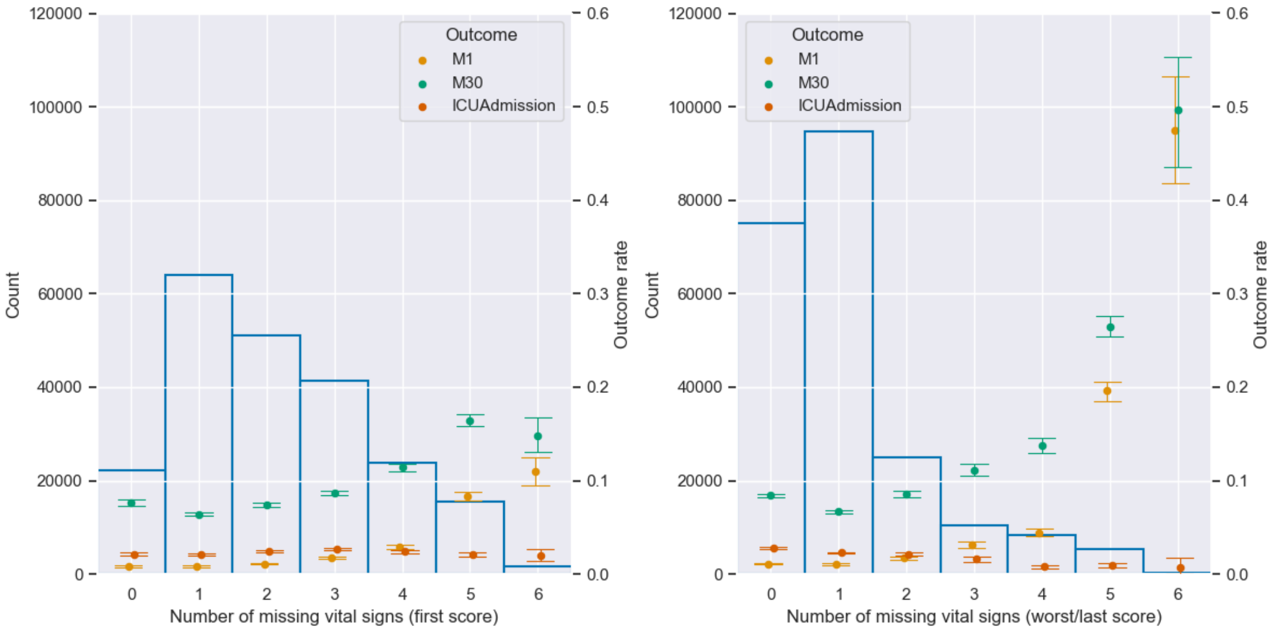

© 2023 Lindskou TA et al. *JAMA Network Open.*

eFigure 3. ROC and Precision-Recall curves for worst (maximum) scores prediction of 1-day mortality (top), 30-day mortality (middle), ICU admission (bottom)

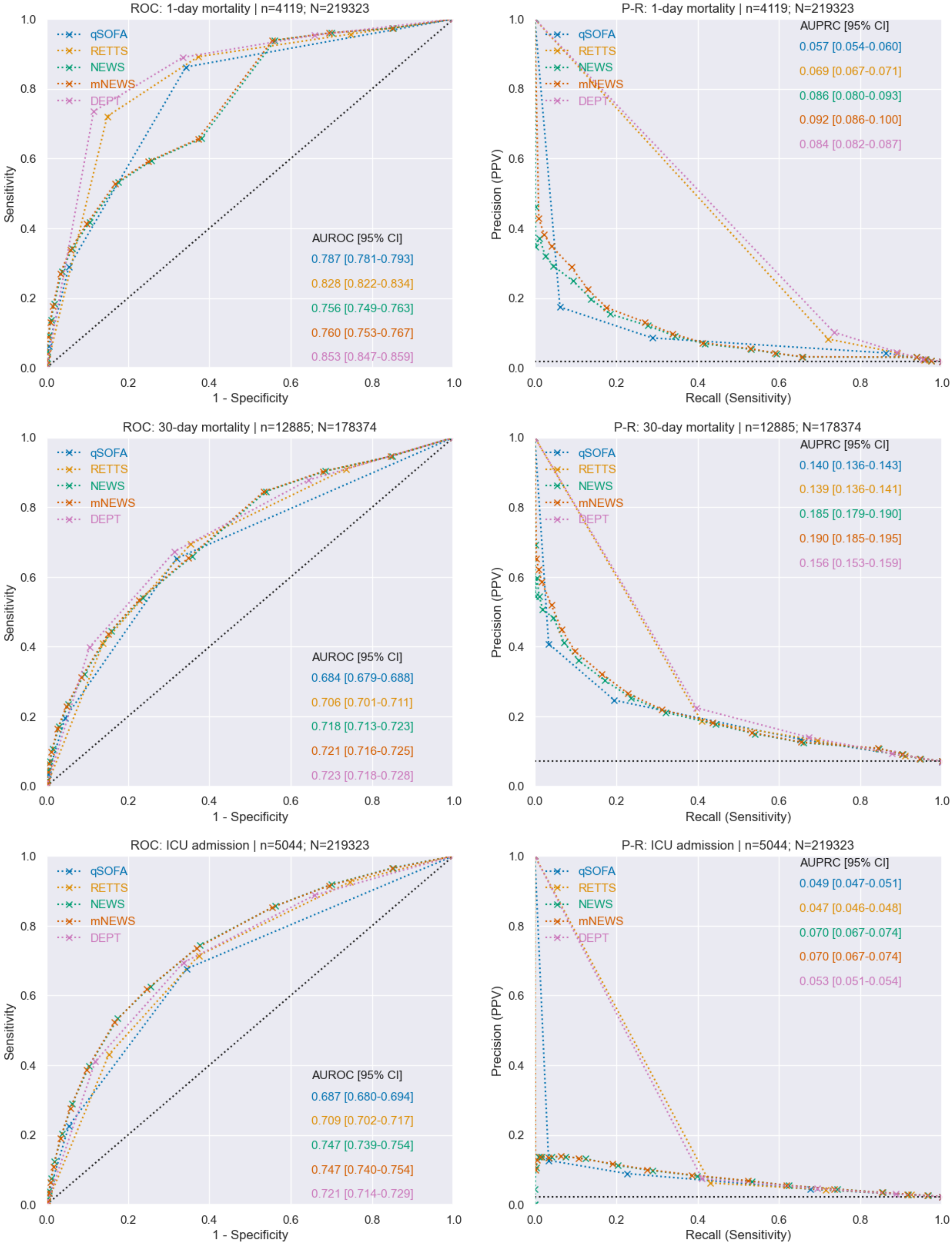

© 2023 Lindskou TA et al. *JAMA Network Open.*

eFigure 4. ROC and Precision-Recall curves for last scores prediction of 1-day mortality (top), 30-day mortality (middle), ICU admission (bottom)

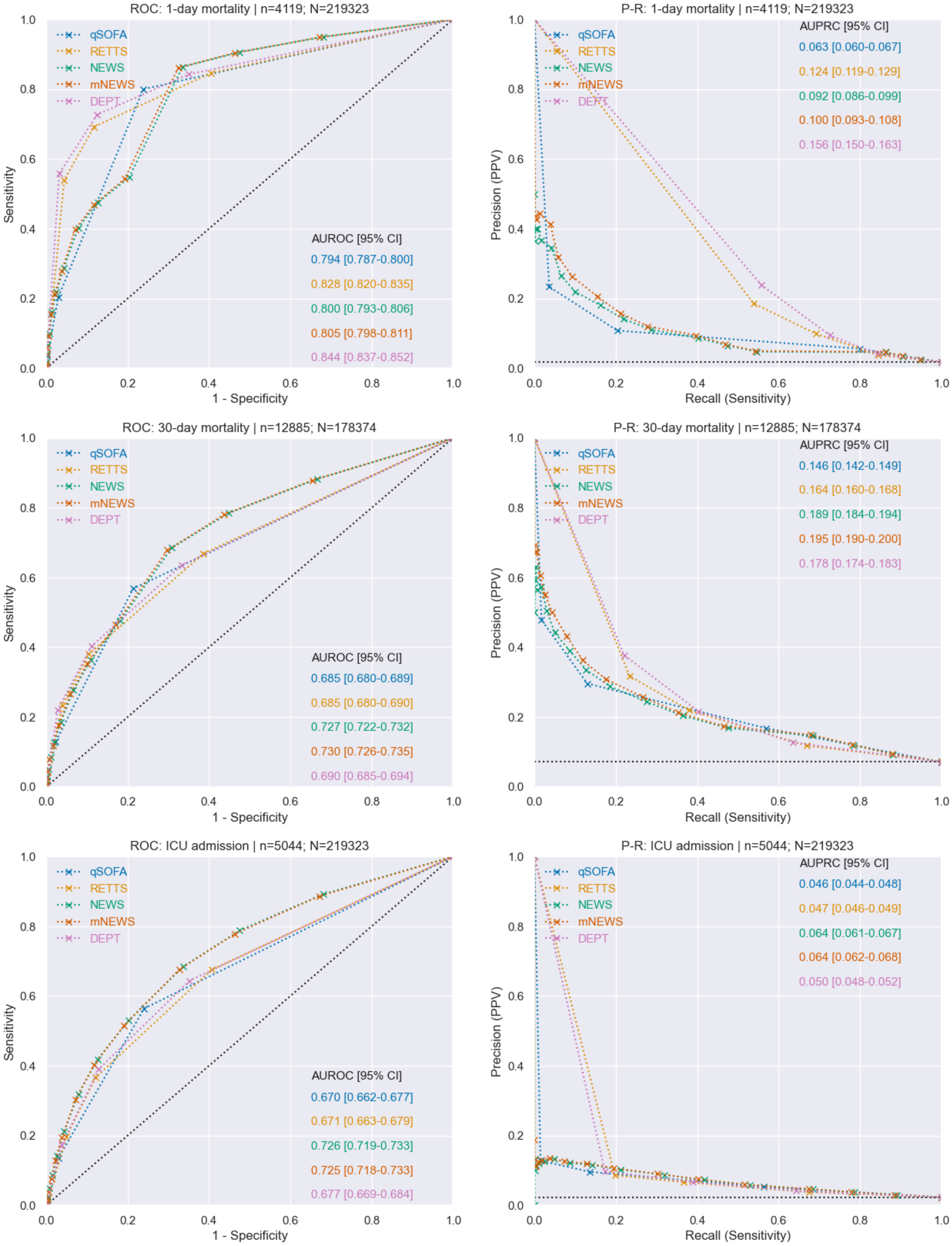

© 2023 Lindskou TA et al. *JAMA Network Open.*

eFigure 5. Sensitivity of EWS predictive ability to minimum number of components used to calculate the score (first score)

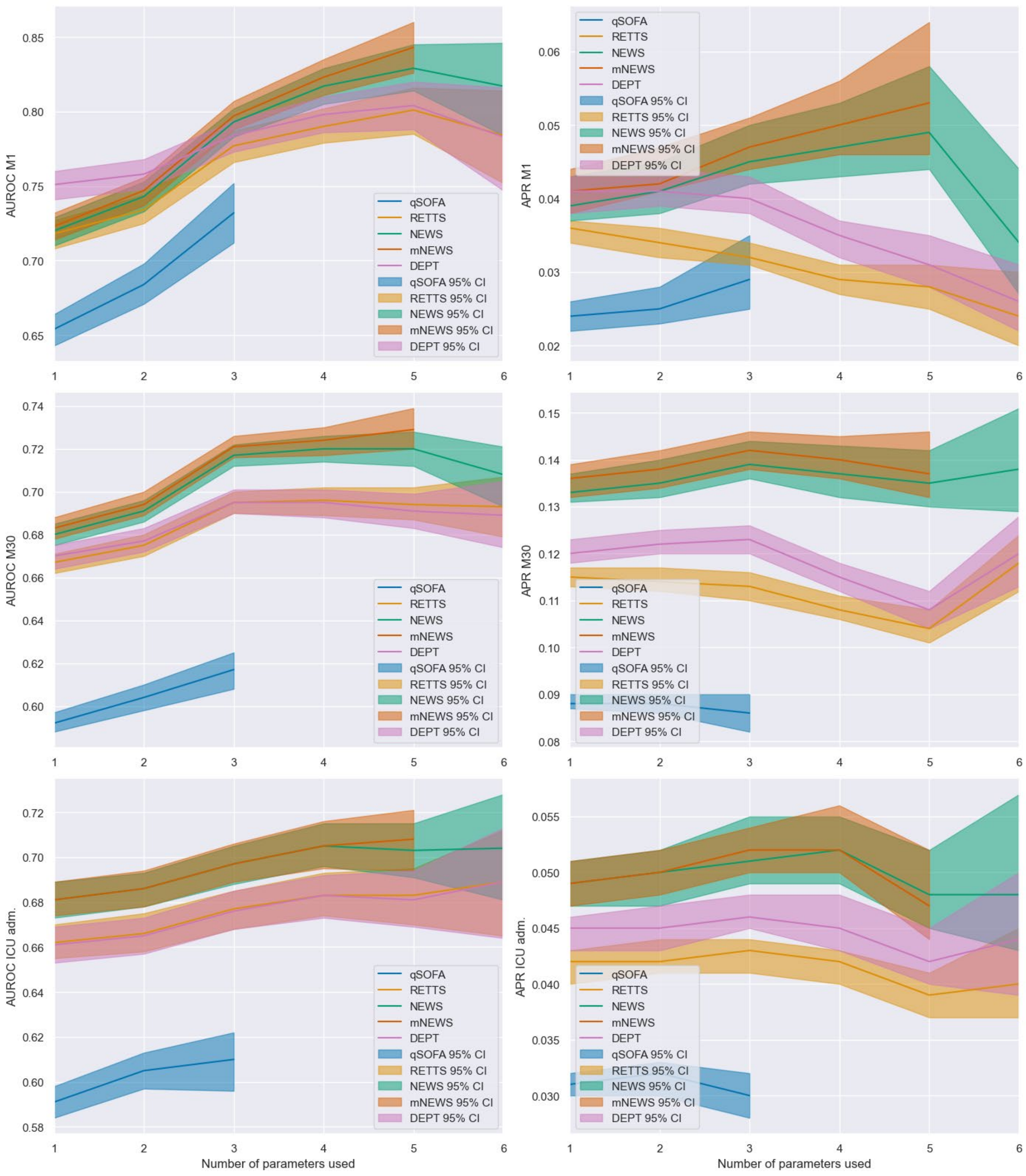

© 2023 Lindskou TA et al. *JAMA Network Open.*

eFigure 6. Sensitivity of EWS predictive ability to minimum number of components used to calculate the score (worst score)

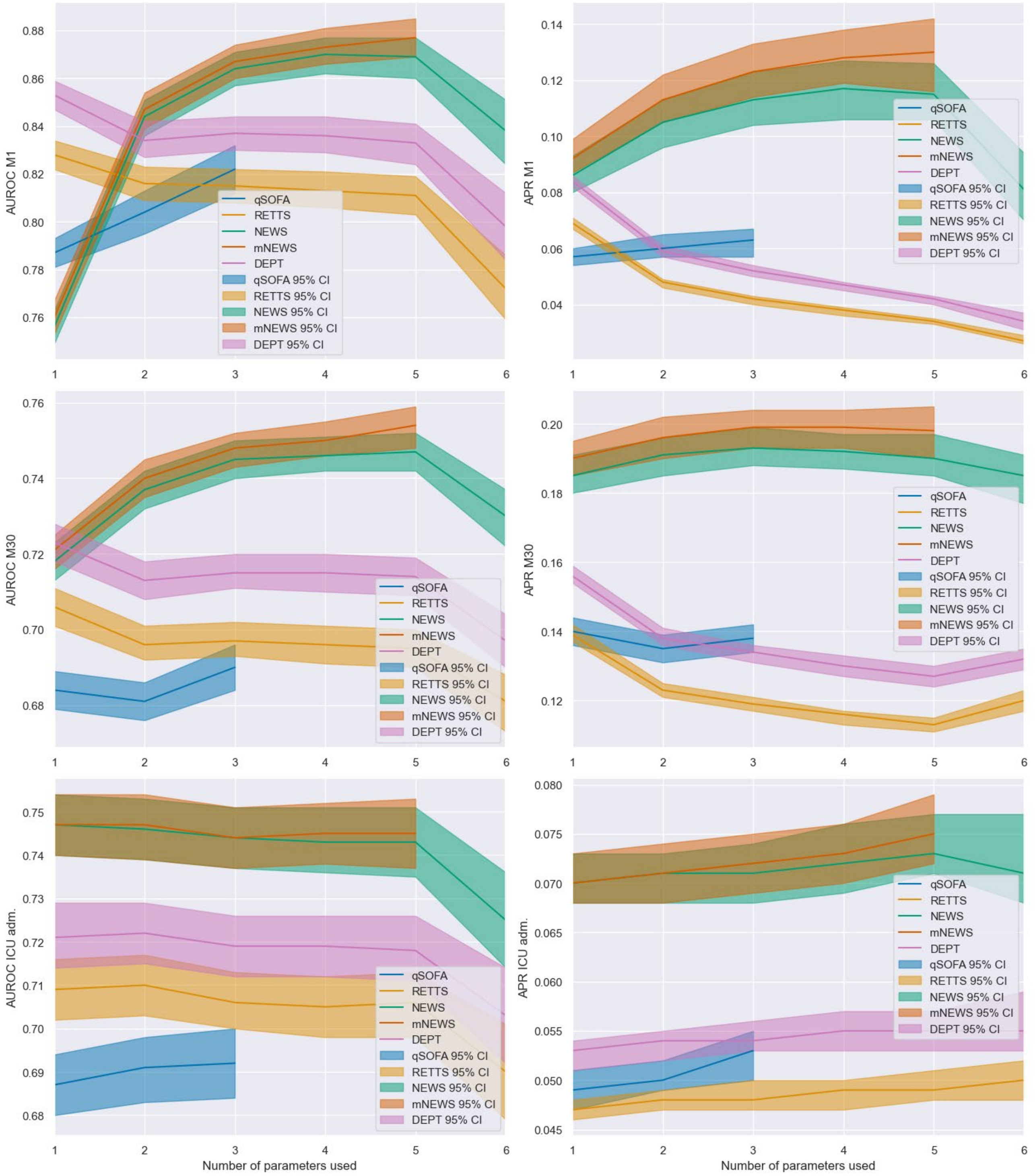

© 2023 Lindskou TA et al. *JAMA Network Open.*

eFigure 7. Sensitivity of EWS predictive ability to minimum number of components used to calculate the score (last score)

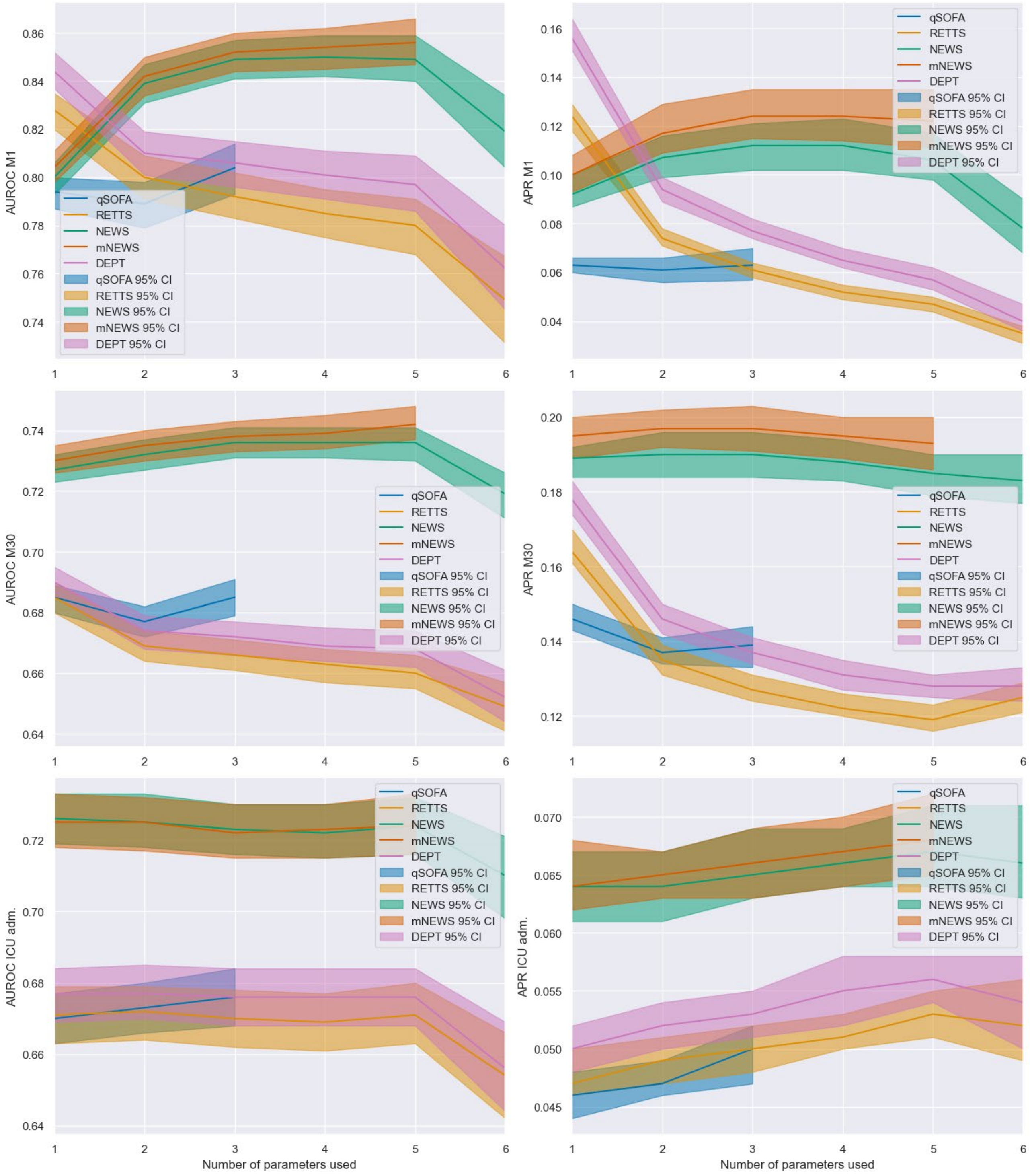

© 2023 Lindskou TA et al. *JAMA Network Open.*
