# Supplementary material for: Prehospital Early Warning Scores to Predict Mortality in Patients Using Ambulances
Source: JAMA Netw Open. 2023 Aug 9;6(8):e2328128. doi: 10.1001/jamanetworkopen.2023.28128 (PMC10413164; doi:10.1001/jamanetworkopen.2023.28128)
Supplement: Supplement 2. — Data Sharing Statement [file jamanetwopen-e2328128-s002.pdf]

Data Sharing Statement

Data
Data available: No

Lindskou. Prehospital Early Warning Scores to Predict Mortality in Patients Using Ambulances. *JAMA Netw Open*. Published online August 9, 2023. doi:10.1001/jamanetworkopen.2023.28128

Additional Information
Explanation for why data not available: Data contain patient sensitive information. Per Danish legislation, approval for handover of patient medical records, may be applied for. If granted, data can then be requested from the Emergency Medical Services, The North Denmark Region
